# Derivation and Validation of a Nomogram for Predicting 90-Day Survival in Patients With HBV-Related Acute-on-Chronic Liver Failure

**DOI:** 10.3389/fmed.2021.692669

**Published:** 2021-06-16

**Authors:** Jun-feng Chen, Wei-zhen Weng, Miao Huang, Xiao-hua Peng, Jian-rong He, Jing Zhang, Jing Xiong, Shao-quan Zhang, Hui-juan Cao, Bin Gao, Deng-na Lin, Juan Gao, Zhi-liang Gao, Bing-liang Lin

**Affiliations:** ^1^Department of Infectious Diseases, Third Affiliated Hospital of Sun Yat-sen University, Guangzhou, China; ^2^Department of Nursing, Guangzhou Red Cross Hospital, Fourth Affiliated Hospital of Jinan University, Guangzhou, China; ^3^Department of Gastroenterology, Seventh Affiliated Hospital of Sun Yat-sen University, Shenzhen, China; ^4^Department of Obstetrics and Gynecology, Green Templeton College, University of Oxford, London, United Kingdom; ^5^Guangdong Provincial Key Laboratory of Liver Disease, The Third Affiliated Hospital of Sun Yat-sen University, Guangzhou, China; ^6^Key Laboratory of Tropical Disease Control (Sun Yat-sen University), Ministry of Education, Guangzhou, Guangdong, China

**Keywords:** hepatitis B virus, acute-on-chronic liver failure, nomogram, prognosis, MELD score, CLIF-C ACLF score, COSSH score, CLIF-C OF score

## Abstract

**Background:** Conventional prognostic models do not fully reflect the severity of hepatitis B virus (HBV)-related acute-on-chronic liver failure (ACLF). This study aimed to establish an effective and convenient nomogram for patients with HBV-related ACLF.

**Methods:** A nomogram was developed based on a retrospective cohort of 1,353 patients treated at the Third Affiliated Hospital of Sun Yat-sen University from January 2010 to June 2016. The predictive accuracy and discriminatory ability of the nomogram were determined by a concordance index (C-index) and calibration curve, and were compared with current scoring systems. The results were validated using an independent retrospective cohort of 669 patients consecutively treated at the same institution from July 2016 to March 2018. This study is registered at ClinicalTrials.gov (NCT03992898).

**Results:** Multivariable analysis of the derivation cohort found that independent predictors of 90-day survival were age, white blood cell (WBC) count, hemoglobin (Hb), aspartate aminotransferase (AST), total bilirubin (TBil), international normalized ratio, serum creatinine (Cr), alpha fetoprotein (AFP), serum sodium (Na), hepatic encephalopathy (HE), pre-existing chronic liver disease(PreLD), and HBV DNA load. All factors were included in the nomogram. The nomogram calibration curve for the probability of 90-day survival indicated that nomogram-based predictions were in good agreement with actual observations. The C-index of the nomogram was 0.790, which was statistically significantly greater than those for the current scoring systems in the derivation cohort (*P* < 0.001). The results were confirmed in the validation cohort.

**Conclusions:** The proposed nomogram is more accurate in predicting the 90-day survival of patients with HBV-related ACLF than current commonly used methods.

## Introduction

Acute-on-chronic liver failure (ACLF) is the acute deterioration of liver function in patients with chronic liver diseases, and the condition progresses rapidly with a mortality rate of more than 50% (1). Liver transplantation is the only treatment that has been proven beneficial for ACLF (2). However, the number of liver donors is limited. As such, development of a simple and accurate prognostic method is necessary to that liver transplantation can be performed on patients with the greatest needed.

Model for end-stage liver disease (MELD) score is commonly used to evaluate patients with ACLF for transplantation (3). However, MELD score only takes into account total bilirubin (TBil), international normalize ratio (INR), and serum creatinine (Cr). Other important valuables such as age, hepatic encephalopathy (HE), and indexes of infection are not included, even though these indexes had been proven to be important for predicting outcomes (4, 5). Furthermore, studies have shown that MELD score does not fully reflect the severity of liver failure (6).

In recent years, the European Association for the Study of the Liver (EASL)-Chronic Liver Failure (CLIF) Consortium Organ Failure (CLIF-C OF) score and CLIF-C ACLF score have been proposed to determine the prognosis of ACLF patients in the West (4, 7). However, the CLIF-C OF score and CLIF-C ACLF score might not be the most appropriate models for predicting the outcomes of Eastern ACLF patients. First, Eastern and Western definitions of ACLF are different. In the West, ACLF is defined as the acute deterioration of patients with cirrhosis (7), while in the East ACLF is considered to develop in both chronic hepatitis and cirrhosis patients (8). In addition, cirrhosis has been confirmed to be an independent risk factor for ACLF (9). Second, most cases of ACLF in the West are associated with alcoholic hepatitis and bacterial infection (7), while in the East most are associated with reaction of hepatitis B virus (HBV) and alcohol abuse (8, 10).

In 2017, Li et al. (5) reported a new prognostic scoring system (Chinese Group on the study of Severe Hepatitis B, COSSH ACLF score) specifically for HBV-ACLF, and the method was superior to MELD, Model for end-stage liver disease with the addition of the Na level (MELD-Na), Child-Turcotte-Pugh (CTP), CLIF-C OF, and CLIF-C ACLF scores. However, the COSSH ACLF score system only includes the INR, TBil, age, Cr, HE, mean arterial pressure, and respiratory function. It is difficult to predict the complex progress of HBV-ACLF comprehensively, meanwhile, it is not convenient to use in clinical practice because of the need for complicated calculations. Thus, it would be necessary to establish an effective and more convenient prognostic scoring system for HBV-ACLF patients.

Nomograms are graphical depictions of predictive statistical models, and have advantages over traditional scoring systems with respect to predicting outcomes (11). Nomograms have been developed and used for various diseases, including liver failure (12, 13). They have also been developed to guide treatment allocation for critical diseases (14).

Thus, the purpose of this study was to develop a nomogram using clinical and laboratory factors to predict 90-day survival in patients with HBV-ACLF, and to compare the predictive value with traditional scoring methods.

## Materials and Methods

### Study Design and Participants

This was a retrospective cohort study performed at a single center in southern China. Consecutive HBV-ACLF patients treated at the Third Affiliated Hospital of Sun Yat-sen University from January 2010 to June 2016 were included for model development (derivation cohort; *n* = 1,353). An second, independent cohort of HBV-ACLF patients with the same inclusion and exclusion criteria treated at the same institution from July 2016 to March 2018 were included to validate the model (validation cohort; *n* = 669).

Inclusion criteria for the study were based on the consensus recommendations of the Asian Pacific Association for the Study of the Liver (APASL) 2014 (10), and Diagnostic and Treatment Guidelines for Liver Failure in China (15). The study inclusion criteria were: (1) ACLF, characterized by acute hepatic deterioration manifesting as jaundice (TBil ≥ 10× the upper limit of normal, in micromoles per liter) and coagulopathy (INR ≥ 1.5, or prothrombin activity < 40%), complicated within 4 weeks by ascites and/or encephalopathy in patients with previously diagnosed or undiagnosed chronic liver disease; (2) Positive serum HBV surface antigen (HBsAg) for more than 6 months; (3) Age > 18 years. Exclusion criteria were: (1) Systemic or local malignancy; (2) HIV infection or other immunodeficiency disease; (3) hepatitis C virus (HCV) infection; (4) Marked organ dysfunction (e.g., renal dysfunction) not related to liver disease (detailed definitions are presented in the Supplementary Material); (5) Pregnancy or lactation; (6) Incomplete data or lost to follow-up; (7) Hospital stay < 1 day.

All patients received comprehensive medical treatment as required, including nutritional supplementation, administration of human serum albumin (ALB), and appropriate treatment for complications such as infections (e.g., respiratory tract, urinary tract, biliary tract, digestive tract, and spontaneous peritonitis), HE, gastrointestinal bleeding (GB), and hepatorenal syndrome (HRS).

This study conformed strictly to the Ethical Guidelines of the 1975 Declaration of Helsinki. The study protocol was approved by the Ethics Committee on Clinical Trials of the Third Affiliated Hospital of Sun Yat-sen University. Due to the retrospective nature of the study, informed consent was waived. This study is registered at ClinicalTrials.gov (NCT03992898).

### Follow-Up and Outcome Measures

Patients were followed up for 90 days, until death, or until liver transplantation. Survival and transplantation data were collected from medical records or by contacting the patient or their family members. The study endpoint was 90-day transplantation-free survival.

### Potential Predictors

After enrollment, demographic and clinical and laboratory data were collected using the hospital information system. Data collected were patient sex and age, precipitating event(s), blood pressure (BP), and pulse oximetry data. Laboratory data included white blood cell (WBC) count, hemoglobin (Hb) level, platelet (PLT) count, serum ALB, globulin, serum sodium (Na), alanine aminotransferase (ALT), aspartate aminotransferase (AST), TBil, INR, serum Cr, alpha fetoprotein (AFP), HBsAg, HBV e antigen (HBeAg), HBV e antibody (HBeAb), and HBV DNA. Antiviral treatments for HBV (nucleoside analogs, including lamivudine, adefovir, entecavir, telbivudine, and tenofovir) within 6 months prior to and during hospitalization were recorded. Complications of ACLF examined included ascites, HE, HRS, GB, and infection. Pre-existing chronic liver diseases (PreLD), including hepatitis and cirrhosis (detailed definitions are presented in the Supplementary Material), were recorded.

### Model Derivation

Continuous variables were transformed into categorical variables based on clinical, routine cutoff points. The cutoff points for AFP and HBV DNA were based on quartile and median, respectively. Survival curves were produced using the Kaplan-Meier method, and compared by the log-rank test. Risk factors associated with 90-day transplantation-free survival were examined in the derivation cohort using Cox proportional hazards models. Variables with values of *P* < 0.05 in the univariate Cox regression analysis included in the multivariate analysis using backward stepwise selection (Entry: 0.05, Removal: 0.1). Data were presented with hazard ratios (HR) and 95% confidence intervals (CIs).

A nomogram (HBV-ACLF nomogram) was formulated based on the results of multivariable Cox regression analyses. The performance of the HBV-ACLF nomogram was evaluated by Harrell's concordance index (C-index), and assessed by comparing nomogram-predicted vs. observed Kaplan-Meier estimates of survival probability; bootstraps with 1,000 resamples were applied to these analyses.

### Model Validation

The total points of each patient in the validation cohort were calculated according to the established HBV-ACLF nomogram, and then Cox regression in this cohort was performed using the total points as a factor. Finally, the C-index and calibration curve were derived based on the regression analyses.

### External Validation of Current Scoring Systems

Comparisons between the HBV-ACLF nomogram and MELD score, MELD-Na score, and CTP score were performed in the derivation and validation cohorts, and with CLIF-C OF, CLIF-C ACLF, and COSSH ACLF scores in the validation cohort. Comparisons were performed with the rcorrp.cens function in the Hmisc package in R software (16). Comparisons were tested using the C-index: a greater C-index indicates more accurate prognostic stratification.

All related programs that are part of R and were used for creating the nomogram are described in detail in the Supplementary Material. Statistical analyses to identify risk factors for 90-day transplantation-free survival were performed using SPSS version 22.0 for Windows (SPSS Inc., Chicago, IL). The HBV-ACLF nomogram was computed with the rms package in R, version 3.6.1 (http://www.r-project.org/) (17). All statistical tests were two-sided, and *P*-values of < 0.05 were considered to be statistically significant.

## Results

### Profile and General Characteristics at Baseline

**Figure 1:**
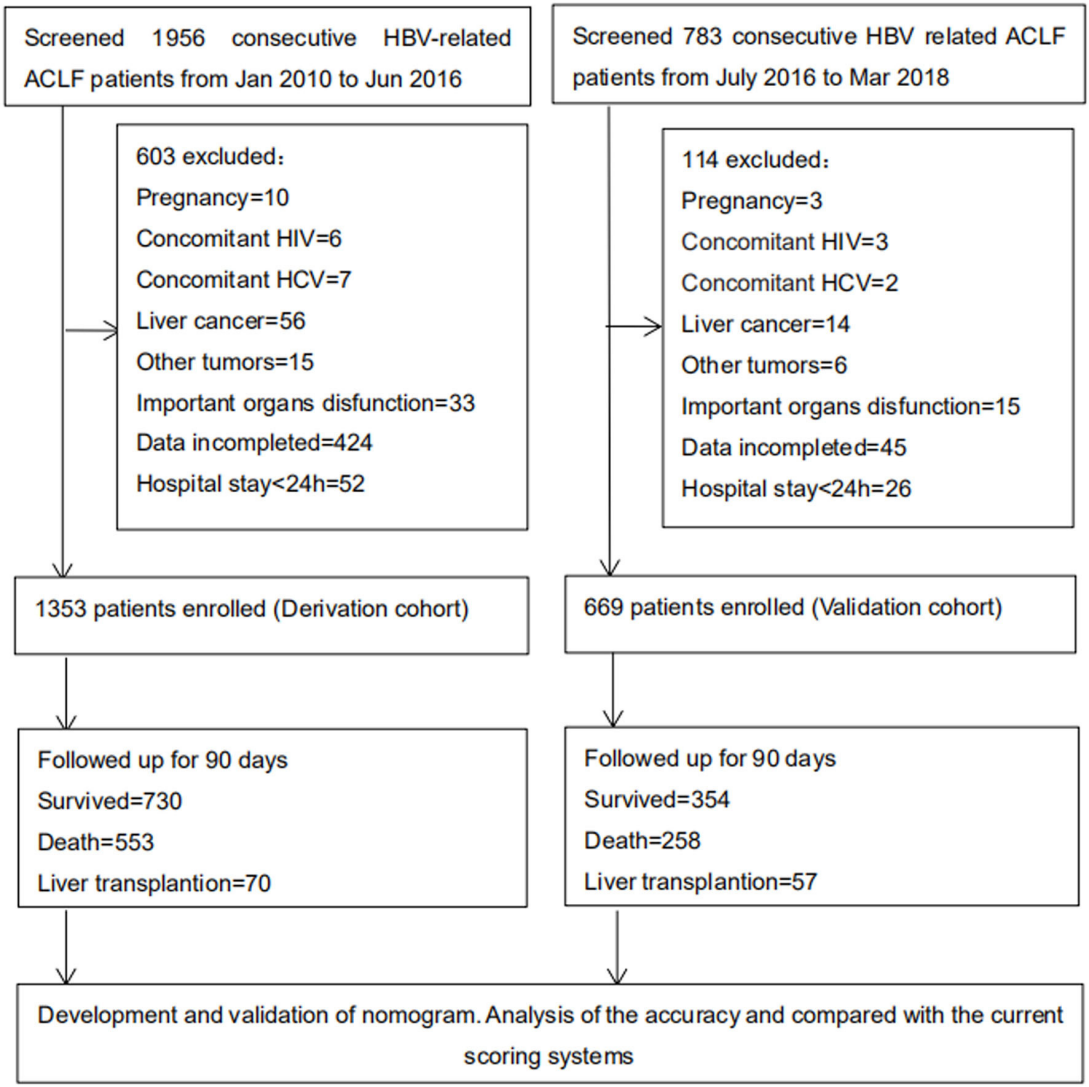
Study profile. A total of 1,956 (from January 2010 to June 2016) and 783 (from July 2016 to March 2018) consecutive HBV-related ACLF patients were screened as for derivation and validation cohorts. Finally 1,353 and 669 patients were included. And 730 and 354 patients were follow-up for 90 days, respectively. HBV, hepatitis B virus; ACLF, acute-on-chronic liver failure; HIV, human immunodeficiency virus; HCV, hepatitis C virus.

A total of 1,956 and 783 consecutive HBV-ACLF patients were screened as for inclusion in the derivation and validation cohorts, respectively. Of these, 603 and 114, respectively, were excluded. Thus, 1,353 patients were included in the derivation cohort, and 669 patients were included in the validation cohort (Figure 1). Patient baseline clinical and laboratory data of the derivation and validation cohorts are shown in Table 1 (Continuous data prior to transformation into categorical variables are shown in Supplementary Table 1).

**Table 1:** Patient demographics and clinical characteristics^†^.

**Characteristic**	**Derivation cohort**	**Validation cohort**	***P*-value**
	**(*n* = 1,353) (No. %)**	**(*n* = 669) (No. %)**	
Age, years			<0.001
18–29	132(9.8)	52(7.8)	
30–39	365(27.0)	141(21.1)	
40–49	399(29.5)	191(28.6)	
50–59	283(20.9)	161(24.1)	
≥60	174(12.9)	124(18.5)	
Sex			0.009
Male	1,221(90.2)	578(86.4)	
Female	132(9.8)	91(13.6)	
WBC, 10^9^/L			0.587
<4	94(6.9)	55(8.2)	
4–10	950(70.2)	464(69.4)	
>10	309(22.8)	150(22.4)	
Hb, g/L			0.235
Male: <120, Female: <110	569(42.1)	308(46.0)	
Male:120–160, Female:110–150	764(56.5)	352(52.6)	
Male: >160, Female: >150	20(1.5)	9(1.3)	
PLT, 10^9^/L			0.002
<100	521(38.5)	294(43.9)	
100–300	820(60.6)	360(53.8)	
>300	12(0.9)	15(2.2)	
ALT, U/L			0.520
<200	522(38.6)	269(40.2)	
200–799	452(33.4)	229(34.2)	
≥800	379(28.0)	171(25.6)	
AST, U/L			0.856
<200	607(44.9)	295(44.1)	
200–799	537(39.7)	274(41.0)	
≥800	209(15.4)	100(14.9)	
ALB, g/L			0.003
<28	200(14.8)	77(11.5)	
28–34.9	799(59.1)	371(55.5)	
≥35	354(26.2)	221(33.0)	
TBil, μmol/L			0.003
171–256.5	256(18.9)	147(22.0)	
256.6–342.0	287(21.2)	171(25.6)	
342.1–427.5	281(20.8)	149(22.3)	
427.6–513.0	242(17.9)	93(13.9)	
>513.0	287(21.2)	109(16.3)	
INR			0.365
1.5–1.99	372(27.5)	188(28.1)	
2.0–2.49	365(27.0)	194(29.0)	
2.5–2.99	233(17.2)	122(18.2)	
≥3.0	383(28.3)	165(24.7)	
Na, mmol/L			0.002
<135	416(30.7)	170(25.4)	
135–145	929(68.7)	487(72.8)	
>145	8(0.6)	12(1.8)	
Cr, μmol/L			0.805
<1ULN	1,248(92.2)	611(91.3)	
1–1.49ULN	67(5.0)	34(5.1)	
1.5–1.99ULN	20(1.5)	12(1.8)	
≥2.0ULN	18(1.3)	12(1.8)	
AFP, ng/ml			0.237
<15.19	338(25.0)	157(23.5)	
15.19–50.12	338(25.0)	180(26.9)	
50.13–149.83	339(25.1)	186(27.8)	
>149.83	338(25.0)	146(21.8)	
HBeAg			0.193
Positive	437(32.3)	197(29.4)	
Negative	916(67.7)	472(70.6)	
HBV DNA, IU/ml			0.289
<149,000	678(50.1)	352(52.6)	
≥149,000	675(49.9)	317(47.4)	
Pre-existing chronic liver diseases			0.011
Chronic hepatitis	575(42.5)	245(36.6)	
Cirrhosis	778(57.5)	424(63.4)	
Alcoholic liver disease			0.992
Yes	105(7.8)	52(7.8)	
No	1,248(92.2)	617(92.2)	
Potential precipitating events			<0.001
Bacterial infection	53(3.9)	38(5.7)	
Re-activation or flare of HBV	653(48.3)	394(58.9)	
Super-infection of hepatitis E virus	90(6.7)	43(6.4)	
Hyperthyroidism	27(2.0)	9(1.3)	
Hepatotoxic drugs	66(4.9)	41(6.1)	
Alcohol	73(5.4)	34(5.1)	
Unknown	391(28.9)	110(16.4)	
Hepatorenal syndrome			0.217
Yes	51(3.8)	33(4.9)	
No	1,302(96.2)	636(95.1)	
Hepatic encephalopathy			0.182
None	1,076(79.5)	555(83.0)	
Grade 1–2	234(17.3)	97(14.5)	
Grade 3–4	43(3.2)	17(2.5)	
Gastrointestinal bleeding			0.176
Yes	16(1.2)	13(1.9)	
No	1,337(98.8)	656(98.1)	
Infection			0.456
Yes	986(72.9)	477(71.3)	
No	367(27.1)	192(28.7)	
MELD score			0.054
<20	97(7.2)	68(10.2)	
20–30	928(68.6)	452(67.6)	
>30	328(24.2)	148(22.1)	
MELD-Na score			0.023
<20	81(6.0)	56(8.4)	
20–30	818(60.5)	421(62.9)	
>30	454(33.6)	192(28.7)	
CTP score			0.021
5–6	0(0)	0(0)	
7–9	245(18.1)	150(22.4)	
10–15	1,108(81.9)	519(77.6)	
Antivirus drug			<0.001
None	125(9.2)	39(5.8)	
LAM	59(4.4)	4(0.6)	
ADV	7(0.5)	0(0.0)	
ETV	1,052(77.8)	556(83.1)	
TDF	26(1.9)	46(6.9)	
Ldt	27(2.0)	3(0.4)	
Combination therapy	57(4.2)	21(3.1)	

† *Clinical and biochemical data were expressed as No. (%)*.

‡ *Alcohol liver disease is defined according to the guideline of prevention and treatment for alcoholic liver disease (2018, China) (18)*.

*WBC, white blood cell; Hb, hemoglobin; PLT, platelet; ALT, alanine aminotransferase; AST, glutamic-oxaloacetic transaminase; ALB, albumin; TBil, total bilirubin; INR, international normalized ratio; Na, serum sodiun; Cr, serum creatinine; AFP, alpha fetal protein; HBeAg, hepatitis B e antigen; HBV, hepatitis B virus; MELD, model for end-stage liver disease; MELD-Na, Model for End-Stage Liver Disease with the addition of the Na level; CTP, Child-Turcotte-Pugh; LAM, lamivudine; ADV, adefovir dipivoxil; ETV, entecavir; TDF, tenofovir; Ldt, telbivudine*.

### Survival Rate and Risk Factors Associated With 90-Day Transplantation-Free Survival in the Derivation Cohort

The 90-day transplantation-free survival rate of patients in the derivation cohort was 54.0%. Univariate analysis indicated that age, WBC count, Hb, PLT, AST, ALB, TBil, INR, Na, Cr, AFP, HBeAg, HBV DNA, HRS, GB, HE, infection, and PreLD were associated with 90-day transplantation-free survival (see Supplementary Table 2). Multivariable analyses demonstrated that age, WBC count, Hb, AST, TBil, INR, Cr, Na, AFP, HBV DNA, HE, and PreLD were independent risk factors for 90-day transplantation-free survival (Table 2).

**Table 2:** Multivariable analysis of the derivation cohort^†^.

	**90 Days' survival**		
**Variable**	**HR**	**95%CI**	***P* value**
Age, years			<0.001
18–29	Reference		
30–39	1.60	1.04–2.45	0.031
40–49	1.72	1.33–2.24	<0.001
50–59	1.81	1.44–2.28	<0.001
≥60	2.58	2.05–3.25	<0.001
WBC, 10^9^/L			0.007
<4	Reference		
4–10	0.78	0.57–1.08	0.134
>10	1.18	0.93–1.50	0.174
Hb, g/L			0.007
Male: <120, Female: <110	Reference		
Male:120–160, Female:110–150	1.31	1.08–1.59	0.006
Male: >160, Female: >150	1.74	0.93–3.25	0.081
AST, U/L			<0.001
<200	Reference		
200–799	1.36	1.11–1.67	0.003
≥800	1.75	1.38–2.22	<0.001
TBil, μmol/L			<0.001
171–256.5	Reference		
256.6–342	1.35	1.01–1.81	0.046
342.1–427.5	1.44	1.13–1.83	0.003
427.6–513	1.88	1.50–2.36	<0.001
>513	1.70	1.39–2.07	<0.001
INR			<0.001
1.5–1.99	Reference		
2.0–2.49	1.15	0.88–1.51	0.316
2.5–2.99	1.97	1.57–2.48	<0.001
≥3.0	2.41	2.02–2.89	<0.001
Na, mmol/L			0.052
<135	Reference		
135–145	0.81	0.68–0.98	0.026
>145	1.44	0.66–3.15	0.360
Cr, μmol/L			<0.001
<1ULN	Reference		
1–1.49ULN	0.98	0.87–1.65	0.899
1.5–1.99ULN	1.84	1.32–3.63	0.027
≥2.0ULN	4.65	2.00–5.72	<0.001
AFP, ng/ml			<0.001
<15.19	Reference		
15.19–50.12	0.87	0.70–1.08	0.198
50.13–149.83	0.68	0.55–0.83	<0.001
>149.83	0.48	0.38–0.62	<0.001
HBV DNA, IU/ml			0.005
<149,000	Reference		
≥149,000	1.32	1.09–1.60	0.005
Pre-existing chronic liver diseases			0.001
Chronic hepatitis	Reference		
Cirrhosis	1.38	1.13–1.69	0.001
Hepatic encephalopathy			<0.001
None	Reference		
Grade 1–2	1.58	1.29–1.95	<0.001
Grade 3–4	2.82	1.90–4.18	<0.001

*CI, confidence interval; HR, hazard ratio; WBC, white blood cell; Hb, hemoglobin; AST, glutamic-oxaloacetic transaminase; TBil, total bilirubin; INR, international normalized ratio; Na, serum sodiun; Cr, serum creatinine; AFP, alpha fetal protein; HBV, hepatitis B virus*.

† *Hazard ratios estimated by Cox proportional hazards regression. All statistical tests were two-sided*.

### Establishing HBV-ACLF Predictive Nomogram

**Figure 2:**
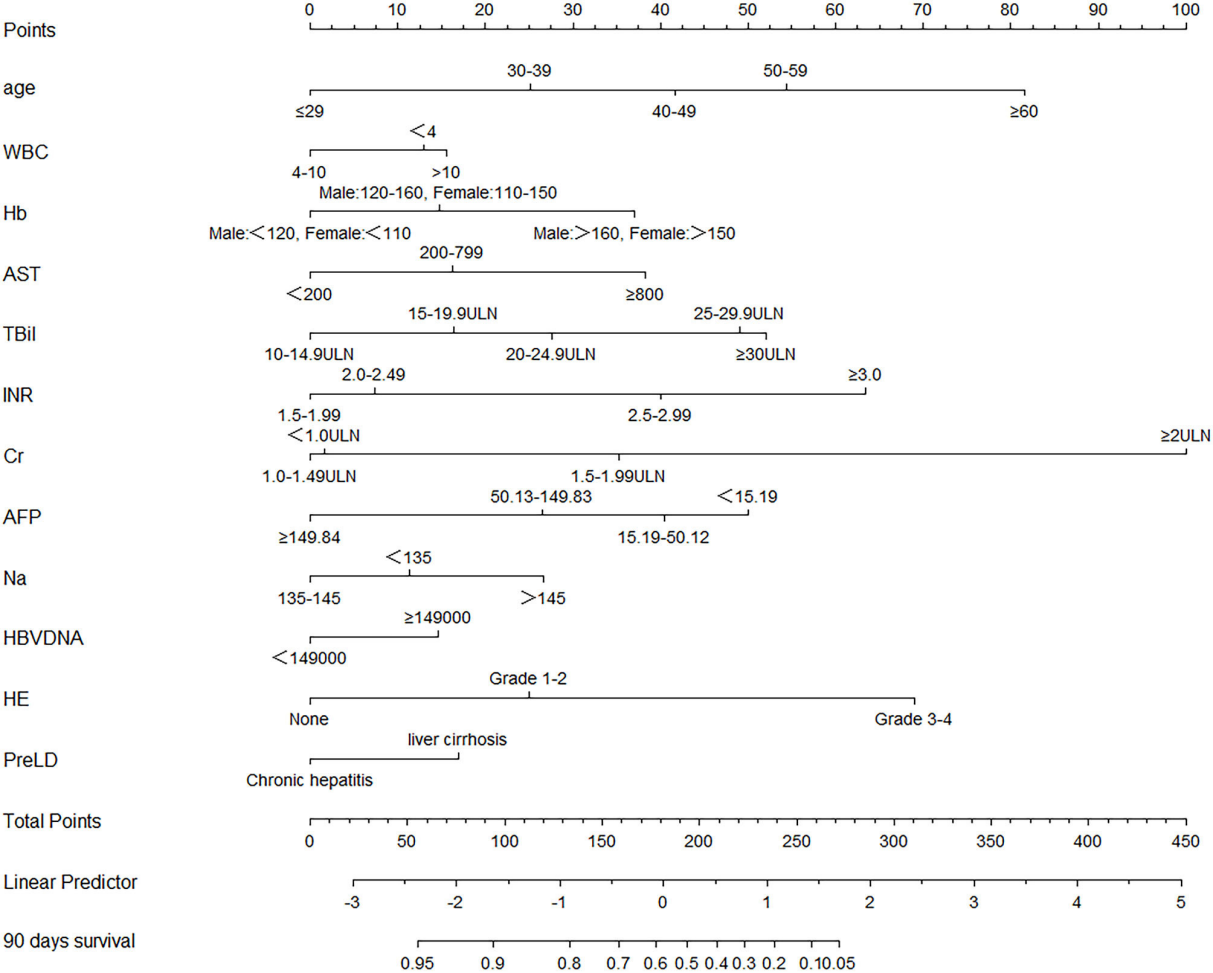
Nomogram, including age, WBC count, Hb, AST, TBil, INR, Cr, AFP, Na, HBV DNA, HE, and PreLD for 90-day transplantation-free survival in HBV-related ACLF patients. The nomogram allows the user to obtain the probability of 90 days transplantation-free survival corresponding to a patient's combination of covariates. As an example, locate the patient's TBil and draw a line straight upward to the “Points” axis to determine the score associated with that TBil. Repeat the process for each variable, and sum the scores achieved for each covariate, and locate this sum on the “Total Points” axis. Draw a line straight down to determine the likelihood of 90 days transplantation-free survival. WBC, white blood cell; Hb, hemoglobin; AST, glutamic-oxaloacetic transaminase; TBil, total bilirubin; INR, international normalized ratio; Cr, serum creatinine; AFP, alpha fetal protein; Na, serum sodium; HBV, hepatitis B virus; HE, hepatic encephalopathy; PreLD, pre-existing chronic liver diseases; ACLF, acute-on-chronic liver failure.

**Figure 3:**
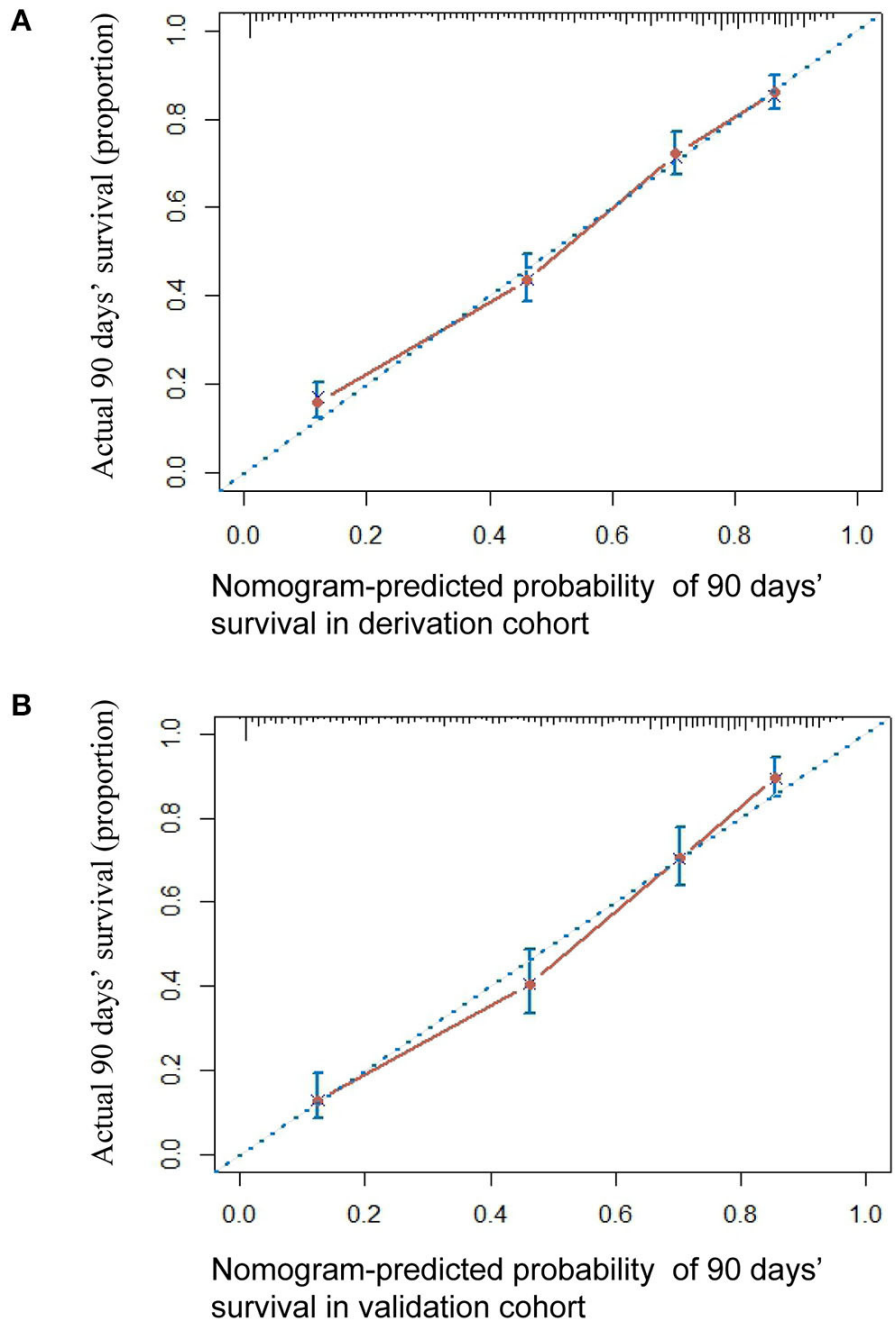
The calibration curve of nomogram for predicting 90-day transplantation-free survival in the derivation cohort **(A)** and in the validation cohort **(B)**. Actual probability of 90-day transplantation-free survival is plotted on the y-axis; nomogram-predicted probability of 90-day transplantation-free survival is plotted on the x-axis.

The HBV-ACLF nomogram to predict 90-day transplantation-free survival of patients with HBV-related ACLF was developed using the variables of age, WBC count, Hb, AST, TBil, INR, Cr, Na, AFP, HBV DNA, HE, and PreLD (Figure 2). The calibration plots for the 90-day transplantation-free survival rate showed optimal agreement between nomogram prediction and actual observation in the derivation cohort (Figure 3A).

### Nomogram Validation

The 90-day transplantation-free survival rate of the validation cohort was 52.9%. The C-index of the HBV-ACLF nomogram for predicting 90-day transplantation-free survival in the validation cohort was 0.793 (95% CI: 0.770–0.817). A calibration curve showed good agreement between predicted 90-day transplantation-free survival using the nomogram and observed survival based on the Kaplan-Meier method (Figure 3B).

### Comparison of Predictive Accuracy Between the Nomogram and CTP, MELD, and MELD-Na Scores in the Derivation and Validation Cohorts

The predictive results for 90-day transplantation-free survival of the HBV-ACLF nomogram and CTP, MELD, and MELD-Na scores are shown in Table 3. In the derivation cohort, the C-index of HBV-ACLF nomogram was 0.790 (95% CI: 0.773–0.807), which was greater than the C-index of the CTP score (0.627; 95% CI: 0.606–0.647, *P* < 0.001), MELD score (0.717; 95% CI: 0.697–0.736, *P* < 0.001), and MELD Na score (0.709; 95% CI: 0.689–0.728, *P* < 0.001).

**Table 3:** The C-index of HBV-related ACLF-Nomogram and different scoring systems for prediction of 90 days' survival in the derivation and validation cohorts.

	**Derivation cohort**		**Validation cohort**	
**Factor**	**C-index (95%CI)**	***P-*value^†^**	**C-index (95%CI)**	***P*-value^†^**
HBV-ACLF-Nomogram	0.790 (0.773,0.807)		0.793 (0.770,0.817)	
MELD	0.717 (0.697,0.736)		0.712 (0.682,0.741)	
MELD-Na	0.709 (0.689,0.728)		0.715 (0.686,0.744)	
CTP	0.627 (0.606,0.647)		0.629 (0.598,0.659)	
CLIF-C OF			0.727 (0.701,0.754)	
CLIF-C ACLF			0.746 (0.720,0.772)	
COSSH ACLF			0.762 (0.737,0.787)	
HBV-ACLF-Nomogram vs. MELD		<0.001		<0.001
HBV-ACLF-Nomogram vs. MELD-Na		<0.001		<0.001
HBV-ACLF-Nomogram vs. CTP		<0.001		<0.001
HBV-ACLF-Nomogram vs. CLIF-C OF				<0.001
HBV-ACLF-Nomogram vs. CLIF-C ACLF				0.001
HBV-ACLF-Nomogram vs. COSSH ACLF				0.002

*C-index, concordance index; HBV, hepatitis B virus; ACLF, acute on chronic liver failure; CI, confidence interval; MELD, model for end-stage liver disease; MELD-Na, model for end-stage liver disease with the addition of the Na level; CTP, child-Turcotte-Pugh; CLIF-C OF, CLIF-consortium organ failure score; CLIF-C ACLF, CLIF-consortium acute-on-chronic liver failure score; COSSH ACLF, Chinese group on the study of severe hepatitis B acute-on-chronic liver failure score*.

† *P-values are calculated based on normal approximation using function rcorrp.cens in Hmisc package*.

In validation cohort, the C-index of HBV-ACLF nomogram was 0.793 (95% CI: 0.770–0.817). This value was greater than the C-index of the CTP score (0.629; 95% CI: 0.598–0.659, *P* < 0.001), MELD score (0.712; 95% CI: 0.682–0.741, *P* < 0.001), and MELD-Na score (0.715; 95% CI: 0.686–0.744, *P* < 0.001).

The C-index of the CLIF-C OF score was 0.727 (95% CI: 0.701–0.754, *P* < 0.001), of the CLIF-C ACLF score was 0.746 (95% CI: 0.720–0.772, *P* = 0.001), and of the COSSH ACLF score was 0.762 (95% CI: 0.737–0.787, *P* = 0.002) (Table 3). All C-index values were less than that of the HBV-ACLF nomogram.

## Discussion

Acute-on-chronic liver failure is a severe disease that results in multiple organ failure, and has a high short-term mortality. An accurate predictive model is necessary to make clinical decision and prioritize patients for liver transplantation. In this study we developed a nomogram to predict 90-day transplantation-free survival that included age, WBC count, and 10 other risk factors. The HBV-ACLF predictive nomogram developed exhibited better predictive accuracy than current conventional and mainstream models including CTP, MELD, MELD-Na, CLIF-C OF, CLIF-C ACLF, and COSSH ACLF scores.

In addition to common risk factors associated with survival in patients with liver disease, such as TBil, INR, Cr, Na, and HE (6, 9, 19), the nomogram developed in this study included age, AST, AFP, PreLD, HBV DNA, WBC count, and Hb. Among them, AFP is an important indicator of regeneration ability of liver cells. Patients with high AFP levels had a better prognosis than those with low levels, which is similar to the results reported by Singh et al. where a rising AFP level was considered to be associated with translation-free survival (20). Aspartate aminotransferase mainly exists in the mitochondria of cells, and an elevation of AST indirectly reflects mitochondrial damage, and may indicate more serious damage than elevation of ALT. Age was included in our model as in other models (4–6, 9), and likely reflects the risks of comorbidities which increase with age.

In our study, cirrhosis was considered a risk factor for evaluating the prognosis of HBV-ACLF patients. Patients with cirrhosis before the onset of ACLF had worse outcomes than those without cirrhosis, which is consistent with results reported by Wu et al. (9) The regeneration ability of liver cells is insufficient in patients with cirrhosis (21), and the patients with cirrhosis in our study had a greater risk of complications (e.g., HE, HRS, and infection; see Supplementary Table 3) than patients without cirrhosis. Moreover, the cirrhosis patients had worse liver function than the chronic hepatitis patients (see Supplementary Table 3), which may lead to a higher risk of a poor outcome. Therefore, the inclusion of cirrhosis as a risk factor in our model suggested that the nomogram was an appropriate model for Eastern ACLF patients.

HBV DNA load was included in the HBV-ACLF nomogram. We divided ACLF patients into two groups using the median value of the HBV DNA loads (149,000 IU/ml), and patients with loads above the median had a worse prognosis than patients with values below the median. To our knowledge, this is the first time that a scoring system included HBV DNA load. Recently, some studies have indicated that infection is an important factor that leads to the development, and promotes the progression of ACLF (22). The peripheral blood WBC count is a very important indicator of infection, and was included in the nomogram developed in this study. Other studies have indicated that WBC count is predictive of survival in patients with ACLF (4, 23).

The predictive accuracy of the HBV-ACLF nomogram was better than that of CTP, MELD, MELD-Na scores in both the derivation and validation cohort. CTP, MELD, and MELD-Na scores are primarily used to determine the prognosis of cirrhosis patients (3). However, important indexes associated with outcomes such as age, AFP, and infection are not included in these models. HBV-ACLF represents a complex condition that is different from liver cirrhosis in many aspects, such as short-term and long-term survival (24). The nomogram is more accurate than the other methods presumably because it includes more known risk factors.

The CLIF-C OF, CLIF-C ACLF, and COSSH ACLF scoring systems include evaluation of respiratory function using PaO_2_/FiO_2_ or SpO_2_/FiO_2_. However, at our center routine evaluation of respiratory function of ACLF patients with pulse oximetry or arterial blood gas analysis was not begun until July 2016. As such, CLIF-C OF, CLIF-C ACLF, and COSSH ACLF scores could only be calculated for patients in validation cohort. Based on the C-indices, the HBV-ACLF nomogram demonstrated better predictive accuracy than the other three methods. This suggests that the HBV-ACLF nomogram is more suitable for evaluating HBV-ACLF patients in Asia diagnosed by APASL and Chinese guideline criteria and reducing the risk of local bleeding due to arterial blood collecting. We can speculate the reasons for this finding. First, the main cause of ACLF in Asia is HBV infection while in the West is alcohol abuse. Disease progression and outcomes of HBV-induced liver disease and alcohol-induced liver disease are different. Second, non-cirrhosis patients are included in the Eastern diagnostic criteria, while in EASL and AASLD criteria the presence of cirrhosis is a requirement for the diagnosis of ACLF. Third, although the COSSH ACLF scoring system exhibited a good predictive ability (C-index = 0.762) for HBV-ACLF in our study, it does not include the variables of Na, AFP, HBV DNA, and the PreLD (cirrhosis or chronic hepatitis). These variables were proven to be important risk factors influencing the prognosis of patients in this study, and in prior studies (9, 10).

Five related studies had been carried out to evaluate the prognosis of ACLF patients with nomogram (13, 25–28). The relatively small number of cases in these studies led to some important risk factors not being included in the models (such as liver cirrhosis, HBV DNA load, etc.). In addition, four out of five studies only compared the nomograms with conventional models, but not with the current mainstream prognostic scoring systems, such as CLIF-C ACLF score and COSSH ACLF score (13, 25, 27, 28). The model of this study was based on the large cohort data, which could reduce the bias caused by insufficient sample size. At the same time, this study also compared the prediction model with the existing mainstream scoring systems. These are the advantages of this study.

This study had several limitations that should be considered. Firstly, specific patient comorbidities were not included in the nomogram development. Severe comorbidities can affect patient survival. Although study has demonstrated that comorbidities are correlated with the survival of cirrhosis patients (29), it is difficult to create categorized variables and to quantify risk because of the diversity of comorbidities. Secondly, this is a retrospective study and subject to all standard limitations associated with this format. For example, a total of 448 patients were lost to follow-up (without outcome data), which may cause bias for the results. Thirdly, The data were obtained exclusively from one center. Thus, the results need to be validated in other data sets.

In conclusion, we developed and validated a nomogram for predicting 90-day transplantation-free survival in patients with HBV-ACLF. The nomogram is more accurate than current predictive models, including CTP, MELD, MELD Na, CLIF-C OF, CLIF-C ACLF, and COSSH ACLF scores, and is very user-friendly. The nomogram may be useful for allocating medical resources in patients with HBV-ACLF; however, to generalize the use of this nomogram validation with data from other institutions is required.

## Data Availability Statement

The datasets presented in this article are not readily available because because of patients' privacy. Requests to access the datasets should be directed to linbingl@mail.sysu.edu.cn.

## Ethics Statement

The studies involving human participants were reviewed and approved by Ethics Committee on Clinical Trials of the Third Affiliated Hospital of Sun Yat-sen University. Written informed consent for participation was not required for this study in accordance with the national legislation and the institutional requirements.

## Author Contributions

B-lL and J-fC designed the study, J-fC and J-rH performed the analysis and interpretation of the data. J-fC, W-zW, MH, X-hP, S-qZ, H-jC, JZ, JX, BG, D-nL, and JG participated in the data collection and follow-up of patients, B-lL and Z-lG provided financial support for this work, J-fC and B-lL wrote and edited the manuscript. All authors contributed to the article and approved the submitted version.

## Conflict of Interest

The authors declare that the research was conducted in the absence of any commercial or financial relationships that could be construed as a potential conflict of interest.
