# Supplementary material for: Derivation and Validation of a Nomogram for Predicting 90-Day Survival in Patients With HBV-Related Acute-on-Chronic Liver Failure
Source: Front Med (Lausanne). 2021 Jun 16;8:692669. doi: 10.3389/fmed.2021.692669 (PMC8241917; doi:10.3389/fmed.2021.692669)
Supplement: Supplementary file 1 [file Table_1.DOCX]

| **Supplementary Table 1. Patients’ continuous variable of demographics and clinical characteristics**^†^ | | |
| --- | --- | --- |
| **Characteristic** | **Derivation cohort**  **(n=1353)** | **Validation cohort**  **(n=669)** |
| Age,y | 44（36, 53） | 46(38, 56) |
| WBC, 10^9^/L | 7.42（5.76, 9.78） | 7.35(5.50, 9.64) |
| Hb, g/L | 123（107, 136） | 120(106, 134) |
| PLT, 10^9^/L | 113（78, 151） | 108(72, 151) |
| ALT, U/L | 346（107.0, 905.5） | 343(103, 812) |
| AST,U/L | 235(118.5, 543.5) | 230(113.0, 534.0) |
| ALB, g/L | 32.1（29.5, 35.1） | 33.10(29.95, 35.95) |
| TBil, µmol/L | 379.3（283.3, 487.8） | 349.5(264.8, 458.55) |
| INR | 2.39（1.96, 3.14） | 2.35(1.94, 2.98) |
| Na, mmol/L | 137（134, 139） | 137(134.65, 139.05) |
| Cr, µmol/L | 70（61, 83） | 71(62, 84) |
| AFP, ng/mL | 50.13（15.19, 149.84） | 49.88(16.44, 134.69) |
| HBV DNA, IU/mL(log10) | 5.22（3.67, 6.70） | 5.05(3.53, 6.70) |
| MELD score | 25.64(22.74, 29.74) | 25.57(22.61, 29.43) |
| MELD-Na score | 26.67(23.38, 32.13) | 26.21(23.07, 30.98) |
| CTP score | 11(10, 12) | 10(10, 11) |
| CILF-C OF score | - | 8(7, 9) |
| CLIF-C ACLF score | - | 38.47(33.71, 43.86) |
| COSSH ACLF score | - | 5.83(5.22, 6.63) |

^†^Clinical and biochemical data were expressed as median and interquartile range. WBC, white blood cell; Hb, hemoglobin; PLT, platelet; ALT, alanine aminotransferase; AST, glutamic-oxaloacetic transaminase; ALB, albumin; TBil, total bilirubin; INR, international normalized ratio; Na, serum sodiun; Cr, serum creatinine; AFP, alpha fetal protein; MELD, Model for End-Stage Liver Disease; MELD-Na, Model for End-Stage Liver Disease with the addition of the Na level; CTP, Child-Turcotte-Pugh; CLIF-C OF, CLIF-Consortium Organ Failure Score; CLIF-C ACLF, CLIF-Consortium Acute-on-chronic Liver Failure Score; COSSH ACLF, Chinese Group on the Study of Severe Hepatitis B Acute-on-chronic Liver Failure Score.

| **Supplementary Table 2. Univariate analysis of the derivation cohort**^†^ | | | |
| --- | --- | --- | --- |
|  | **90 days’ survival** | | |
| **Variable** | **HR** | **95%CI** | **P** |
| Age,y  18-29  30-39  40-49  50-59  ≥60 | Reference  1.64  1.93  1.98  2.78 | 1.09-2.46  1.50-2.47  1.60-2.45  2.26-3.42 | ＜0.001  0.017  ＜0.001  ＜0.001  ＜0.001 |
| Sex  Male  Female | Reference  1.19 | 0.92-1.53 | 0.184  0.184 |
| WBC, 10^9^/L  ＜4  4-10  ＞10 | Reference  0.80  1.52 | 0.59-1.08  1.23-1.88 | ＜0.001  0.151  ＜0.001 |
| Hb, g/L  Male:＜120, Female:＜110  Male:120-160, Female:110-150  Male:＞160, Female:＞150 | Reference  0.80  1.25 | 0.68-0.94  0.69-2.28 | 0.017  0.006  0.457 |
| PLT, 10^9^/L  ＜100  100-300  ＞300 | Reference  0.62  0.47 | 0.53-0.73  0.15-1.47 | ＜0.001  ＜0.001  0.196 |
| AST, U/L  ＜200  200-799  ≥800 | Reference  1.19  1.61 | 1.00-1.41  1.32-1.96 | ＜0.001  0.056  ＜0.001 |
| ALB, g/L  ＜28  28-34.9  ≥35 | Reference  0.81  0.63 | 0.65-1.00  0.52-0.77 | ＜0.001  0.046  ＜0.001 |
| TBil, µmol/L  171-256.5  256.6-342  342.1-427.5  427.6-513  >513 | Reference  1.36  1.41  1.44  1.72 | 1.02-1.80  1.13-1.76  1.16-1.77  1.45-2.05 | ＜0.001  0.034  0.003  0.001  ＜0.001 |
| INR  1.5-1.99  2.0-2.49  2.5-2.99  ≥3.0 | Reference  1.25  2.18  3.51 | 0.96-1.63  3.00-4.17  3.99-4.11 | ＜0.001  0.105  ＜0.001  ＜0.001 |
| Na, mmol/L  ＜135  135-145  ＞145 | Reference  0.52  2.97 | 0.44-0.61  1.41-6.25 | ＜0.001  ＜0.001  0.004 |
| Cr, µmol/L  ＜1ULN  1-1.49ULN  1.5-1.99ULN  ≥2.0ULN | Reference  2.18  3.15  4.67 | 1.62-2.94  1.94-5.12  2.79-7.81 | ＜0.001  ＜0.001  ＜0.001  ＜0.001 |
| AFP, ng/ml  <15.19  15.19-50.12  50.13-149.83  >149.83 | Reference  0.82  0.91  0.40 | 0.67-1.01  0.50-0.74  0.32-0.50 | ＜0.001  0.061  ＜0.001  ＜0.001 |
| HBeAg  Negative  Positive | Reference  0.76 | 0.64-0.91 | 0.002  0.002 |
| HBV DNA, IU/ml  ＜149000  ≥149000 | Reference  1.21 | 1.03-1.41 | 0.019  0.019 |
| Pre-existing chronic liver diseases  Chronic hepatitis  Cirrhosis | Reference  1.92 | 1.62-2.28 | ＜0.001  ＜0.001 |
| Hepatorenal Syndrome  No  Yes | Reference  4.16 | 3.07-5.65 | ＜0.001  ＜0.001 |
| Hepatic Encephalopathy  None  Grade 1-2  Grade 3-4 | Reference  2.65  2.69 | 2.21-3.17  1.89-3.83 | ＜0.001  ＜0.001  ＜0.001 |
| Gastrointestinal Bleeding  No  Yes | Reference  1.89 | 1.04-3.43 | 0.037  0.037 |
| Infection  No  Yes | Reference  1.84 | 1.51-2.25 | ＜0.001  ＜0.001 |

^†^ Hazard ratios estimated by Cox proportional hazards regression. All statistical tests were two-sided. CI, confidence interval; HR, hazard ratio; WBC, white blood cell; Hb, hemoglobin; PLT, platelet; ALT, alanine aminotransferase; AST, glutamic-oxaloacetic transaminase; ALB, albumin; TBil, total bilirubin; INR, international normalized ratio; Na, serum sodiun; Cr, serum creatinine; AFP, alpha fetal protein; HBV, hepatitis B virus; HBeAg, hepatitis B e antigen; HBV, hepatitis B virus.

| **Supplementary Table 3. Comparison of demographics and clinical characteristics between cirrhosis and non-cirrhosis HBV-related ACLF patients in two cohorts**^†^ | | | | | | | |
| --- | --- | --- | --- | --- | --- | --- | --- |
| **Characteristic** | **Derivation cohort** | | |  | **Validation cohort** | | |
|  | **Non-cirrhosis patients**  **(n=575)** | **Cirrhosis patients**  **(n=778)** | **P value** |  | **Non-cirrhosis patients**  **(n=245)** | **Cirrhosis patients**  **(n=424)** | **P value** |
| Age,y | 40(32, 48) | 47(39, 56) | <0.001 |  | 42(35, 49) | 50(41, 59) | <0.001 |
| Sex  Male  Female | 536(93.2%)  39(6.8%) | 685(88.0%)  93(12.0%) | 0.002 |  | 224(91.4%)  21(8.6%) | 354(83.5%)  70(16.5%) | 0.004 |
| WBC, 10^9^/L | 8.06  (6.53, 10.31) | 6.94  (5.05, 9.22) | <0.001 |  | 8.11  (6.50, 10.45) | 6.81  (5.09, 9.26) | <0.001 |
| Hb, g/L | 130  (116, 141) | 116.5  (101.0, 130.4) | <0.001 |  | 128  (115, 140) | 114  (100, 127) | <0.001 |
| PLT, 10^9^/L | 143  (144, 182) | 85  (62, 112) | <0.001 |  | 149.5  (121.5, 184.0) | 84  (59, 112) | <0.001 |
| ALT, U/L | 684  (281, 1288) | 179.0  (77.0, 518.3) | <0.001 |  | 701  (361, 1381) | 160.0  (78.3, 490.8) | <0.001 |
| AST,U/L | 370  (159, 714) | 176.5  (100.0, 378.3) | <0.001 |  | 399.0  (163.5, 809.0) | 189.0  (98.3, 368.5) | <0.001 |
| ALB, g/L | 32.5  (29.8, 35.2) | 32.0  (28.0, 35.0) | 0.014 |  | 34.0  (31.2, 36.4) | 32.4  (29.5, 35.5) | <0.001 |
| TBil, µmol/L | 352.2  (269.0, 459.7) | 408.5  (299.0, 525.4) | <0.001 |  | 334.5  (259.9, 420.3) | 370.2  (272.0, 485.3) | 0.002 |
| INR | 2.26  (1.88, 2.97) | 2.48  (2.01, 3.28) | <0.001 |  | 2.20  (1.84, 2.71) | 2.46  (1.99, 3.12) | <0.001 |
| Na, mmol/L | 137.6  (135.8, 140.0) | 136  (133, 138) | <0.001 |  | 138  (136, 140) | 136.9  (133.1, 139.0) | <0.001 |
| Cr, µmol/L | 69.0  (61.0, 80.0) | 71  (61, 88) | 0.007 |  | 71.0  (62.5, 80.0) | 71.5  (61.0, 89.0) | 0.150 |
| AFP, ng/mL | 80.43  (24.42, 225.10) | 37.13  (10.47, 110.23) | <0.001 |  | 77.78  (28.17, 186.03) | 36.36  (12.38, 101.90) | <0.001 |
| HBeAg  Positive  Negative | 218(37.9%)  357(62.1%) | 219(28.1%)  559(71.9%) | <0.001 |  | 86(35.1%)  159(64.9%) | 111(26.2%)  313(73.8%) | 0.015 |
| HBV DNA, IU/mL(log10) | 5.68  (4.36, 7.19) | 4.79  (3.27, 6.34) | <0.001 |  | 5.95  (4.69, 7.40) | 4.54  (3.16, 6.07) | <0.001 |
| MELD score | 24.84  (22.38, 28.29) | 26.24  (23.17, 30.91) | <0.001 |  | 24.57  (21.88, 27.13) | 26.34  (23.01, 30.49) | <0.001 |
| MELD-Na score | 25.45  (22.68, 29.23) | 28.02  (24.10, 34.37) | <0.001 |  | 24.88  (22.21, 28.26) | 27.46  (23.83, 33.27) | <0.001 |
| CTP score | 10(10, 11) | 11(10, 12) | <0.001 |  | 10(9, 11) | 11(10, 12) | <0.001 |
| CILF-C OF score | - | - | - |  | 8(7, 9) | 9(8, 9) | <0.001 |
| CLIF-C ACLF score | - | - | - |  | 36.48  (32.36, 41.60) | 39.85  (34.90, 45.27) | <0.001 |
| COSSH ACLF score | - | - | - |  | 5.44  (4.99, 6.15) | 6.14  (5.41, 7.01) | <0.001 |
| Alcoholic liver disease^‡^  Yes  No | 35(6.1%)  540(93.9%) | 70(9.0%)  708(91.0%) | 0.048 |  | 19(7.8%)  226(92.2%) | 33(7.8%)  391(92.2%) | 0.990 |
| Potential precipitating events  Bacterial infection  Re-activation or flare of HBV  Super-infection of hepatitis E virus  Hyperthyroidism  Hepatotoxic drugs  Alcohol  Unknown | 17(3.0%)  290(50.4%)  41(7.1%)  15(2.6%)  20(3.5%)  38(6.6%)  154(26.8%) | 36(4.6%)  363(46.7%)  49(6.3%)  12(1.5%)  46(5.9%)  35(4.5%)  237(30.5%) | 0.031 |  | 13(5.3%)  155(63.3%)  22(9.0%)  2(0.8%)  14(5.7%)  19(7.8%)  20(8.2%) | 25(5.9%)  239(56.4%)  21(5.0%)  7(1.7%)  27(6.4%)  15(3.5%)  90(21.2%) | <0.001 |
| Ascite  Yes  No | 320(55.7%)  255(44.3%) | 534(68.7%)  244(31.3%) | <0.001 |  | 92(37.6%)  153(62.4%) | 278(65.6%)  146(34.4%) | <0.001 |
| Hepatorenal Syndrome  Yes  No | 9(1.6%)  566(98.4%) | 42(5.3%)  736(94.6%) | <0.001 |  | 3(1.2%)  242(98.8%) | 30(7.1%)  394(92.9%) | 0.001 |
| Hepatic Encephalopathy  None  Grade 1-2  Grade 3-4 | 472(82.1%)  81(14.1%)  22(3.8%) | 604(77.6%)  153(19.7%)  21(2.7%) | 0.017 |  | 215(87.8%)  23(9.4%)  7(2.8%) | 340(80.2%)  74(17.5%)  10(2.4%) | 0.017 |
| Gastrointestinal Bleeding  Yes  No | 5(0.9%)  570(99.1%) | 11(1.4%)  767(98.6%) | 0.360 |  | 0(0%)  245(100%) | 13(3.1%)  411(96.9%) | 0.003 |
| Infection  Yes  No | 380(67.0%)  195(33.0%) | 606(77.9%)  172(22.1%) | <0.001 |  | 154(62.9%)  91(37.1%) | 323(76.2%)  101(23.8%) | <0.001 |

^†^Clinical and biochemical data were expressed as No. (%), median and interquartile range.

^‡^Alcohol liver disease is defined according to the guideline of prevention and treatment for alcoholic liver disease(2018, China).^18^

WBC, white blood cell; Hb, hemoglobin; PLT, platelet; ALT, alanine aminotransferase; AST, glutamic-oxaloacetic transaminase; ALB, albumin; TBil, total bilirubin; INR, international normalized ratio; Na, serum sodiun; Cr, serum creatinine; AFP, alpha fetal protein; HBeAg, hepatitis B e antigen; HBV, hepatitis B virus; MELD, Model for End-Stage Liver Disease; MELD-Na, Model for End-Stage Liver Disease with the addition of the Na level; CTP, Child-Turcotte-Pugh；CLIF-C OF, CLIF-Consortium Organ Failure Score; CLIF-C ACLF, CLIF-Consortium Acute-on-chronic Liver Failure Score; COSSH ACLF, Chinese Group on the Study of Severe Hepatitis B Acute-on-chronic Liver Failure Score.
