# Supplementary material for: Derivation and Validation of a Nomogram for Predicting 90-Day Survival in Patients With HBV-Related Acute-on-Chronic Liver Failure
Source: Front Med (Lausanne). 2021 Jun 16;8:692669. doi: 10.3389/fmed.2021.692669 (PMC8241917; doi:10.3389/fmed.2021.692669)
Supplement: Supplementary file 2 [file Data_Sheet_1.DOCX]

Supplementary Material

1. **Some definitions in this study.**

**Cardiac dysfunction**: Patients who have history of hypertension and heart diseases suffer symptoms of palpitations, dyspnea, edema and with left ventricle ejection fraction lower than 60%.

**Respiratory dysfunction**: Patients suffer symptoms of dyspnea and have their blood oxygen saturation lower than 95%.

**Cerebral dysfunction**: Patients have cerebrovascular diseases, such as cerebral hemorrhage, cerebral embolism.

**Cirrhosis:** Cirrhosis was diagnosed based on previous liver biopsy results, clinical evidence of previous decompensation and laboratory test results, endoscopy demonstrating esophageal and/or gastric varices, and radiological imaging showing evidence of portal hypertension and/or liver nodularity.

**2.Related Computerized Programs for the Nomogram Using R in This Study**

**For a Cox Proportional Hazards Model**

S<-Surv(time, status)

f <- cph(s ~ age+ WBC+Hb+ AST+ TBil+ INR+ Cr + AFP+Na+ HBVDNA+ HE+ PreLD, x=TRUE, y=TRUE, surv=TRUE,time.inc=90)

**For nomogram**

survival <- Survival(f)

survival1 <- function(x)survival(90,x)

nom <- nomogram (f,fun = list(survival1),

fun.at = c(0.05,seq(0.1,0.9,by=0.1),0.95),

funlabel= '90 days survival')

plot(nom, xfrac=.45)

**For Resampling Validation of the Nomogram**

validate(f, method="boot",B=1000,dxy=T)

**For Computing the C-Index and 95% CI, and for Comparison of the C-Index**

**in Different Models**

f_cindex<-function(f1, f2) {outcome <- data.frame(cindex=numeric(0), c_95_low=numeric(0), c_95_up=numeric(0), p=numeric(0))

f1c <- rcorrcens(Surv(dat$survivaltime,dat$status==1)~predict(f1))

outcome[1,1] <- 1-f1c[1]

outcome[1,2] <- 1-f1c[1] - f1c[4]*1.96/2

outcome[1,3] <- 1-f1c[1] + f1c[4]*1.96/2

f2c <- rcorrcens(Surv(dat$survivaltime,dat$status==1)~predict(f2))

outcome[2,1] <- 1-f2c[1]

outcome[2,2] <- 1-f2c[1] - f2c[4]*1.96/2

outcome[2,3] <- 1-f2c[1] + f2c[4]*1.96/2

fcom <- rcorrp.cens(predict(f1),predict(f2), Surv(dat$survivaltime,dat$status==1))

outcome[3,4] <- 1-pnorm(abs(fcom["C X1"]-fcom["C X2"])/(fcom["S.D."]/2))

rownames(outcome) <- c(substitute(f1),substitute(f2), "compare")

return(outcome)}

f1<-cph(Surv(survivaltime, status==1)~ age+ WBC+Hb+ AST+ TBil+ INR+ Cr + AFP+Na+ HBVDNA+ HE+ PreLD,data = dat, x=TRUE, y=TRUE, surv=TRUE)

f2 <-cph(Surv(survivaltime, status==1) ~different scoring systems, data = dat, x=TRUE, y=TRUE, surv=TRUE)

f_cindex(f1, f2)

**For External Validation of the Nomogram**

f<-cph(S~totalpoint form nomogram, x=TRUE,y=TRUE,surv=TRUE,time.inc=90)

**For the Calibration Curve**

1）In derivation cohort

f1<-cph(Surv(survivaltime,status==1)~age+ WBC+Hb+ AST+ TBil+ INR+ Cr + AFP+Na+ HBVDNA+ HE+ PreLD, x=T,y=T ,surv=T,time.inc=90)

cal1<-calibrate(f1, cmethod="KM",method="boot",u=90,m=320,B=1000)

plot(cal1,lwd=2,lty=1,errbar.col=c(rgb(0,118,192,maxColorValue=255)),xlim=c(0,1),ylim=c(0,1),xlab="Nomogram-Predicted Probability of 90 days survival",ylab="Actual 90 days survival (proportion)",col=c(rgb(192,98,83,maxColorValue=255)))

lines(cal1[,c("mean.predicted","KM")], type="b",lwd=2,col=c(rgb(192,98,83,maxColorValue=255)),pch=16) abline(0,1,lty=3,lwd=2,col=c(rgb(0,118,192,maxColorValue=255)))

1. In validation cohort

f2<-cph(Surv(dat2$survivaltime,dat2$status==1)~predict(f1,dat2), x=T,y=T,surv=T,time.inc=90)

cal1<-calibrate(f2, cmethod="KM",method="boot",u=90,m=160,B=1000)

plot(cal1,lwd=2,lty=1,errbar.col=c(rgb(0,118,192,maxColorValue=255)),xlim=c(0,1),ylim=c(0,1),xlab="Nomogram-Predicted Probability of 90 days survival",ylab="Actual 90 days survival (proportion)",col=c(rgb(192,98,83,maxColorValue=255)))

lines(cal1[,c("mean.predicted","KM")],type="b",lwd=2,col=c(rgb(192,98,83,maxColorValue=255)),pch=16) abline(0,1,lty=3,lwd=2,col=c(rgb(0,118,192,maxColorValue=255)))
